# Radiation-Induced Lung Injury: Prevention, Diagnostics and Therapy in the Era of the COVID-19 Pandemic

**DOI:** 10.3390/jcm11195713

**Published:** 2022-09-27

**Authors:** Lukas Käsmann, Julian Taugner, Alexander Nieto, Claus Belka, Chukwuka Eze, Farkhad Manapov

**Affiliations:** 1 Department of Radiation Oncology, University Hospital, LMU Munich, 81377 Munich, Germany; 2 Comprehensive Pneumology Center Munich (CPC-M), Member of the German Center for Lung Research (DZL), 81377 Munich, Germany; 3 German Cancer Consortium (DKTK), Partner Site Munich, 81377 Munich, Germany

Thoracic radiotherapy (TRT) plays an integral role in the multimodal treatment of lung cancer, breast cancer, esophageal cancer, thymoma and mesothelioma, having been used as either a definitive, neoadjuvant or adjuvant treatment or for palliative intention to achieve symptom control. However, radiation-induced lung injury presenting as radiation pneumonitis (RP) or lung fibrosis (LF) is still a severe and dose-limiting complication of TRT, proving potentially life-threatening [1,2]. Until now, RILI has unfortunately been poorly understood, mainly characterized by an overwhelming cascade of damage-associated molecular patterns (DAMPs), the release of proinflammatory cytokines and chemokines through death and/or senescent epithelial and endothelial cells and the activation of specific immune cells [3,4]. With technical advances, such as stereotactic body radiation therapy (SBRT), volumetric modulated arc therapy (VMAT) or proton radiotherapy, radiation delivery to the tumor and surrounding tissues could be performed more precisely, reducing the incidence of severe pulmonary toxicity. The rates of severe symptomatic RP (≥III) are reported to be <5% after concurrent chemoradiation, followed by durvalumab maintenance treatment for stage III non-small cell lung cancer (NSCLC) based on the PACIFIC trial [5]. However, several real-world studies reported higher rates of RP in elderly (>65 years) and Asian patients; therefore, the early assessment of RP and treatment is important to prevent therapy-related deaths and a decline in these patients’ quality of life (QoL) [6].

Typically, RP occurs within 3 months following the end of TRT (range: 1–6 months) and is diagnosed by clinical symptoms and associated radiological findings. Several grading scales of RP have been established [2]; however, the Radiation Therapy Oncology Group (RTOG) criteria and the Common Terminology Criteria for Adverse Events (CTCAE) are the most used ones in daily clinical practice. A majority of patients do not show any clinical symptoms, such as persistent, dry and nonproductive coughing, dyspnea, mild fever, pleuritic pain and chest discomfort. More importantly, no standard laboratory test can identify or exclude RP [7]. Typically, radiological changes after TRT are ground-glass opacities (GGOs) or/with airspace consolidation that can be observed in an irradiated field, although may occur in other parts of the lung as well [8], with additional features including (reversed) a halo sign, atelectasis, nodule-like pattern, tree-in-bud appearance, ipsilateral or chronic pleural effusion.

Based on the severity of RP, treatment or clinical monitoring should be considered according to national and international guidelines; asymptomatic patients should be observed without further treatment, while the recommended treatment for symptomatic RP is the use of corticosteroids [2]. Therapy should be performed over several weeks or months and slowly tapered [9]. Sudden discontinuation should be avoided in order to prevent the early relapse of RP (rebound phenomenon) with increased severity and a higher risk of developing LF. Prophylactic treatment with antibiotics in RP can be considered for patients at high risk of bacterial infection, for selected patients with cancer-associated bronchial stenosis or for immunocompromised patients. Immunosuppressive treatment can be amended with azathioprine or cyclosporine in order to reduce steroid dosage. Breathing exercises and the inhalation of β-sympathomimetics can be additionally used as supportive treatments. Severe RP (grade ≥III) is treated by administering oxygen, providing assisted ventilation and the prophylaxis of right heart failure. In contrast to RP, a successful treatment for LF has not yet been established. Several additional agents have been investigated to prevent and/or treat RP and LF, such as ACE (angiotensin-converting enzyme) inhibitors and angiotensin-II receptor subtype 1 (AT-1) antagonists, amifostine or pentoxifylline and pamrevlumab or pirfenidone [2]. A randomized phase two trial (NCT02496585) investigated the prophylactic use of nintedanib to prevent RP. However, due to low recruitment and a change in standard treatment based on the promising results of the PACIFIC trial, the trial was terminated prematurely [5,10]. As a result, none of these agents could be recommended to prevent RP or LF.

To date, the integration of SBRT, introduction of image-guided radiation treatment (IGRT), reduction in tumor motion and critical definition of target volumes have been the best strategies for the prevention of RILI [2,11]. Concurrent systemic treatment should only be used if robust evidence through large prospective trials is established, with comorbidities such as interstitial lung disease needing to be considered to estimate the risk of RILI [12]. The longitudinal monitoring of the diffusing capacity for carbon monoxide (DLCO) after TRT could serve as an early predictive marker [13].

In March 2020, the World Health Organization (WHO) declared wide-spread infections of acute respiratory syndrome caused by coronavirus 2 (SARS-CoV-2) as a pandemic [14]. Compared to the general population, cancer patients are at a higher risk of poor outcomes from SARS-CoV-2, with associated mortality having been reported to reach 40% in nonvaccinated lung cancer patients [15]. Pulmonary symptoms of SARS-CoV-2 infection are highly variable, but interstitial pneumonia is the most severe one, proving to be a potentially life-threatening condition due to acute respiratory distress. The pathogenesis of COVID-associated acute and chronic lung pulmonary damage remains mostly unknown [16].

More importantly, SARS-CoV-2 interstitial pneumonia and RP have shown overlapping radiological and clinical characteristics, both of which could result in clinical deterioration with a decline in respiratory status and QoL due to irreversible lung changes [17]. Clinical symptoms are quite similar, with patients presenting with dyspnea, a dry nonproductive cough and mild fever. In the case of SARS-CoV-2, interstitial pneumonia is without temporal association to TRT in contrast to RP which usually occurs in the first 6 months after the end of TRT [18]. The onset of symptomatic RP is slower than SARS-CoV-2 interstitial pneumonia, which could help guide further diagnostics. In both situations, blood samples usually show high C-reactive protein levels with normal serum procalcitonin. Radiological features of interstitial pneumonia caused by SARS-CoV-2 are also similar to early RP, such as GGO. However, RP is usually observed unilaterally and imaging abnormalities correlate to radiation treatment fields, volumes and distribution [19].

To date, combined RP and SARS-CoV-2 interstitial pneumonia has not been described in the literature. Therefore, future prospective studies are needed to investigate the clinical and radiological findings of SARS-CoV-2 infection and RP. Based on the global registry data of the Thoracic Cancers International COVID-19 Collaboration (TERAVOLT) seven major determinants of death have been identified, namely, age, Eastern Cooperative Oncology Group (ECOG) performance status, tumor stage at COVID-19 diagnosis, the development of pneumonia, neutrophil count, C-reactive protein and serum procalcitonin [20]. Further research needs to address additional outcome parameters, as well as the impact of COVID-19 vaccination, which has not yet been evaluated.

In summary, cancer patients, especially with lung cancer, are at high risk of lethal complications from SARS-CoV-2 infection due to immunosuppressive treatments and reduced lung function. It is pivotal to test any patient suspected of having RP for SARS-CoV-2 infection in order to differentiate between the two and guide the determination of the optimal treatment without delay.

## Data Availability

Not applicable.

## References

[1] Jeong B.K., Kim J.H., Jung M.H., Kang K.M., Lee Y.H. (2021). Cytokine Profiles of Non-Small Cell Lung Cancer Patients Treated with Concurrent Chemoradiotherapy with Regards to Radiation Pneumonitis Severity. J. Clin. Med..

[2] Käsmann L., Dietrich A., Staab-Weijnitz C.A., Manapov F., Behr J., Rimner A., Jeremic B., Senan S., De Ruysscher D., Lauber K. (2020). Radiation-induced lung toxicity–cellular and molecular mechanisms of pathogenesis, management, and literature review. Radiat. Oncol..

[3] Citrin D.E., Shankavaram U., Horton J.A., Shield W., Zhao S., Asano H., White A., Sowers A., Thetford A., Chung E.J. (2013). Role of type II pneumocyte senescence in radiation-induced lung fibrosis. J. Natl. Cancer Inst..

[4] Hansel C., Jendrossek V., Klein D. (2020). Cellular Senescence in the Lung: The Central Role of Senescent Epithelial Cells. Int. J. Mol. Sci..

[5] Antonia S.J., Villegas A., Daniel D., Vicente D., Murakami S., Hui R., Yokoi T., Chiappori A., Lee K.H., de Wit M. (2017). Durvalumab after chemoradiotherapy in stage III non–small-cell lung cancer. N. Engl. J. Med..

[6] Wang Y., Zhang T., Huang Y., Li W., Zhao J., Yang Y., Li C., Wang L., Bi N. (2021). Real-world Safety and Efficacy of Consolidation Durvalumab after Chemoradiotherapy for Stage III Non-small-cell Lung Cancer: A Systematic Review and Meta-analysis. Int. J. Radiat. Oncol. Biol. Phys..

[7] Arroyo-Hernández M., Maldonado F., Lozano-Ruiz F., Muñoz-Montaño W., Nuñez-Baez M., Arrieta O. (2021). Radiation-induced lung injury: Current evidence. BMC Pulm. Med..

[8] Thomas R., Chen Y.-H., Hatabu H., Mak R.H., Nishino M. (2020). Radiographic patterns of symptomatic radiation pneumonitis in lung cancer patients: Imaging predictors for clinical severity and outcome. Lung Cancer.

[9] Kauffmann-Guerrero D., Taugner J., Eze C., Käsmann L., Li M., Tufman A., Manapov F. (2021). Clinical Management and Outcome of Grade III Pneumonitis after Chemoradioimmunotherapy for Inoperable Stage III Non-Small Cell Lung Cancer—A Prospective Longitudinal Assessment. Diagnostics.

[10] Dy G.K., Prasad D., Kumar P., Attwood K., Adjei A.A. (2021). A Phase 2 Randomized, Double-Blind, Placebo-Controlled Study Evaluating Nintedanib Versus Placebo as Prophylaxis Against Radiation Pneumonitis in Patients With Unresectable NSCLC Undergoing Chemoradiation Therapy. J. Thorac. Oncol..

[11] Jain V., Berman A.T. (2018). Radiation pneumonitis: Old problem, new tricks. Cancers.

[12] Goodman C.D., Nijman S.F., Senan S., Nossent E.J., Ryerson C.J., Dhaliwal I., Qu X.M., Laba J., Rodrigues G.B., Palma D.A. (2020). A primer on interstitial lung disease and thoracic radiation. J. Thorac. Oncol..

[13] Stana M., Grambozov B., Gaisberger C., Karner J., Ruznic E., Berchtold J., Zellinger B., Moosbrugger R., Studnicka M., Fastner G. (2022). Carbon Monoxide Diffusing Capacity (DLCO) Correlates with CT Morphology after Chemo-Radio-Immunotherapy for Non-Small Cell Lung Cancer Stage III. Diagnostics.

[14] World Health Organization (WHO) Coronavirus Disease 2019 (COVID 19). Situation Report-74. https://www.who.int/docs/default-source/coronaviruse/situation-reports/20200403-sitrep-74-covid-19-mp.pdf?sfvrsn=4e043d03_10.

[15] Kulkarni A.A., Wilson G., Fujioka N., Patel M.R. (2021). Mortality from COVID-19 in patients with lung cancer. J. Cancer Metastasis Treat..

[16] Bösmüller H., Matter M., Fend F., Tzankov A. (2021). The pulmonary pathology of COVID-19. Virchows Arch..

[17] Ippolito E., Fiore M., Greco C., D’Angelillo R.M., Ramella S. (2020). COVID-19 and radiation induced pneumonitis: Overlapping clinical features of different diseases. Radiother. Oncol..

[18] Shaverdian N., Shepherd A.F., Rimner A., Wu A.J., Simone II C.B., Gelblum D.Y., Gomez D.R. (2020). Need for caution in the diagnosis of radiation pneumonitis during the covid-19 pandemic. Adv. Radiat. Oncol..

[19] Zeng Q., Tang C., Deng L., Li S., Liu J., Wang S., Shan H. (2020). Differential diagnosis of COVID-19 pneumonia in cancer patients received radiotherapy. Int. J. Med. Sci..

[20] Whisenant J.G., Baena J., Cortellini A., Huang L.-C., Russo G.L., Porcu L., Wong S.K., Bestvina C.M., Hellmann M.D., Roca E. (2022). A Definitive Prognostication System for Patients With Thoracic Malignancies Diagnosed With Coronavirus Disease 2019: An Update From the TERAVOLT Registry. J. Thorac. Oncol..

